# Spontaneous Prediction Error Generation in Schizophrenia

**DOI:** 10.1371/journal.pone.0037843

**Published:** 2012-05-30

**Authors:** Yuichi Yamashita, Jun Tani

**Affiliations:** Laboratory for Behavior and Dynamic Cognition, RIKEN Brain Science Institute, Wako, Saitama, Japan; Indiana University, United States of America

## Abstract

Goal-directed human behavior is enabled by hierarchically-organized neural systems that process executive commands associated with higher brain areas in response to sensory and motor signals from lower brain areas. Psychiatric diseases and psychotic conditions are postulated to involve disturbances in these hierarchical network interactions, but the mechanism for how aberrant disease signals are generated in networks, and a systems-level framework linking disease signals to specific psychiatric symptoms remains undetermined. In this study, we show that neural networks containing schizophrenia-like deficits can spontaneously generate uncompensated error signals with properties that explain psychiatric disease symptoms, including fictive perception, altered sense of self, and unpredictable behavior. To distinguish dysfunction at the behavioral versus network level, we monitored the interactive behavior of a humanoid robot driven by the network. Mild perturbations in network connectivity resulted in the spontaneous appearance of uncompensated prediction errors and altered interactions within the network without external changes in behavior, correlating to the fictive sensations and agency experienced by episodic disease patients. In contrast, more severe deficits resulted in unstable network dynamics resulting in overt changes in behavior similar to those observed in chronic disease patients. These findings demonstrate that prediction error disequilibrium may represent an intrinsic property of schizophrenic brain networks reporting the severity and variability of disease symptoms. Moreover, these results support a systems-level model for psychiatric disease that features the spontaneous generation of maladaptive signals in hierarchical neural networks.

## Introduction

The complex and diverse cognitive behavior of humans is enabled by the evolution of functional hierarchies in brain networks [1]–[3]. In these hierarchical neural systems, orderly interactions between *top-down* goal-directed processes, associated with prefrontal cortex, and *bottom-up* sensory-driven processes in primary and associative sensorimotor cortices are essential for flexible behavior [4]–[6]. However, while hierarchical neural systems provide significant advantages for adaptive behavior in social environments, their failure to properly develop or maintain precisely aligned signaling of goal-directed behavioral sequences is proposed to result in neuropsychiatric disease symptoms.

Schizophrenia is a psychiatric disease whose symptoms include spontaneous episodic hallucinations, delusions, disturbances of self, and, in more severe cases, disorganized behavior such as repetitive and cataleptic behaviors. Neuroclinical observations suggest schizophrenia is associated with abnormal functioning of the prefrontal cortex and posterior parts of the brain such as the parietal [7], [8] and temporal cortex [9]. However, the diverse symptoms of schizophrenia cannot be explained merely by anatomical or physiological abnormalities in focal regions, but likely have a global, systems-level origin. Based on this rationale, theoretical [10]–[12] and clinical [13] studies have suggested that the basic pathology of schizophrenia may be associated with “functional disconnectivity” in the hierarchical network of the brain, primarily between prefrontal and posterior brain regions. Such network deficits might arise via cellular defects in circuit formation or function [14], [15]. Likewise, studies from the perspective of motor control theory [16], [17] hypothesized that disturbance of self, a core symptom of schizophrenia, arises due to a failure of patients to form appropriate sensory predictions or “forward models” [18] that are essential for skillful behavior. In this view, the impaired forward model results in a mismatch between the forward prediction and actual sensory feedback, resulting in the patient's feeling that his actions are not generated by himself but by some outside force. Yet another line of theory suggests that schizophrenic patients may have disruptions in error/conflict-related signals [19]–[21], which are an important aspect of top-down and bottom-up interactions. However, since the target symptoms and level of explanation for each of these theories are different, existing models of schizophrenia remain fragmentary. More importantly, the mechanism by which disconnected brain networks could produce defective neural network interactions is unknown. Here we show that underconnected neural networks produce aberrant prediction error signals, and, in turn, these defective signals produce changes in the goal-orientation of the network, even in the absence of behavior. Our proposed idea was tested through a series of experiments in which behavioral control mechanisms were implemented by the physical actions of a humanoid robot driven by a hierarchical neural network model that was required to perform goal-directed behaviors via interactions with its environment.

## Results

### System overview: neural network-driven robot

**Figure 1:**
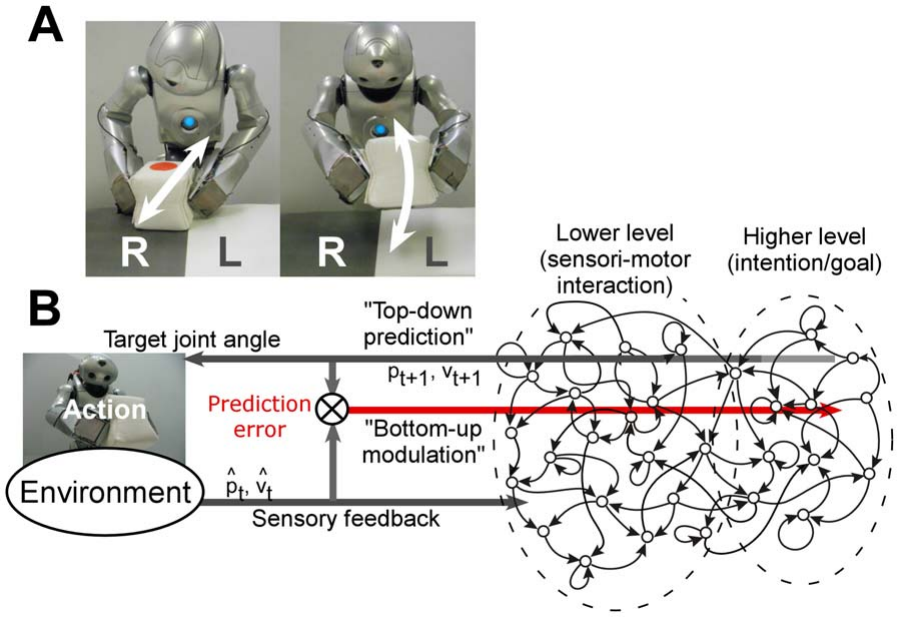
The behavioral task for the robot and system overview. (A) The task for the robot is to repeatedly produce two different types of behavior: (i) move the object up and down three times at the position L, and (ii) move the object backward and forward three times at the position R. For each series of actions, the robot began from the home position and ended at the same home position. The robot repeatedly generates the same series of actions unless the object was located at the same position. The object position was switched by an experimenter at unpredictable timing. (B) System overview.

To study the relationship between neural network activity and goal-directed behavior, we employed a humanoid robot driven by a hierarchical network. The goal of the robot was to repeatedly produce two different types of behavior following a rule associated with the positions of an object (Fig. 1A). In addition to producing a series of action sequences, the robot was also required to flexibly switch between the two types of behavior according to unpredictable changes in its environment under experimental manipulation. In order to achieve such human-like flexible adaptation, the robot must contain an internal neural representation for the current ongoing task and this representation has to be switched for the target task behavior. In this study, this internal representation and corresponding neural activity related to the task behavior are referred to as the “intention/goal” and “intention state”, respectively.

An artificial neural network controlling the robot actions was instantiated by a hierarchical recurrent neural network (RNN) model [3]. Thanks to its capacity to reproduce complex dynamics, the RNN is often used for modeling temporal sequence learning [22]–[25]. Spatio-temporal patterns of behavior arise from dynamics of neural activities through neural connectivity. The RNN is as such considered to emulate characteristic features of actual neural systems, and the current model is considered consistent at the level of the macro-level mechanisms of biological neural systems [24]–[26].

The network receives input from current proprioception and vision sensory modalities, and generates forward predictions of those for the next time step (Fig. 1B). The forward prediction of proprioception was sent to the robot in the form of target joint angles. This forward prediction of sensory states is made possible by the capacity of the RNN to preserve its internal state, which enables it to reproduce complex dynamics. As a result of training, the self-organization of a functional hierarchy occurred, within which one grouping, referred to as a higher level, represented the executive intention/goal for the task behavior, and the other grouping, referred to as a lower level, represented sensorimotor interactions ([3], see also Methods).

### Flexible switching of behavior though hierarchical interactions

Based on this hierarchical representation, the network successfully reproduced learned task behavior sequences as top-down prediction of proprioceptive sequences with the interaction of the robot's body and its physical environment. In addition to top-down forward predictions, in order to achieve quick adaptation to environmental changes, intention states could be modulated based on prediction errors, the discrepancy between the network's prediction and reality (Fig. 1B, see Methods for details). Through the bottom-up modulation process in which the intention state is modulated so as to minimize prediction error [5], [6], the robot successfully adapted to unpredictable sensory perturbations (Movie S1).

**Figure 2:**
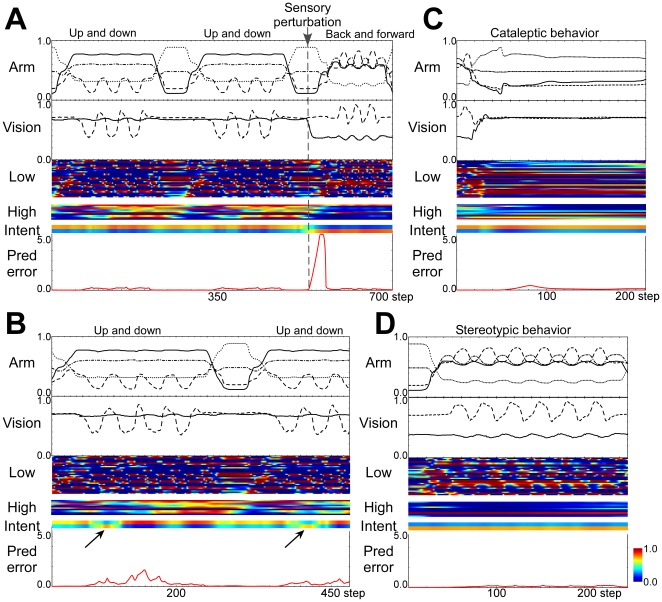
Neural activity and task behavior in normal and disconnected networks. (A) Flexible switching of behavior through the bottom-up modulation process induced by sensory perturbation. (B) Outwardly normal behavior with intermittent increases of prediction error and aberrant modulation of intentional states (arrows) induced by simulated mild functional disconnection in the hierarchical network. (C) Cataleptic and (D) stereotypic behavior induced by the severe disconnection. Arm: 4 dimensional joint angles. Vision: relative position of the object (*x*-*y* axis). A long sideways rectangle indicates the single unit activity over many time steps. Colors of rectangles indicate activation level (cf. color bar). Low and High indicates activity of units in the lower level and the higher level of the network. Intent indicates the activity of parametric bias (PB) units in the higher level, whose activity corresponds to the top-down intention for the task behavior (see Methods). Pred error indicates prediction error accumulating for the past 25 steps.

Figure 2A illustrates an example of sensorimotor sequences and changes in the activity of the trained network during the robot's task execution through real-time interactions between top-down prediction and bottom-up modulation processes. Due to the unpredictable switching of the object's position, prediction error was temporarily increased and this induced modulation of the robot's intention state resulting in the flexible switching of its overt behavior in response to its environment. This switching of intention through bottom-up modulation can be thought of as corresponding to recognition of a situation.

### Simulations of functional network disconnection in schizophrenia

**Figure 3:**
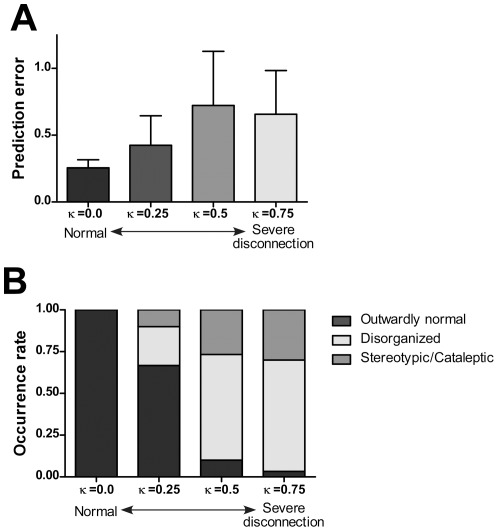
Changes in prediction error and robot behavior associated with levels of disconnection. (A) Prediction error for various levels of disconnection is shown. Bars in the graph correspond to mean values over 30 trials for each parameter setting. Error bars indicate the degree of standard deviation. (B) Changes in robot behavior with various levels of disconnection are shown. Bars in the graph correspond to the occurrence ratio of each behavior type over 30 trials for each parameter setting. Levels of disconnection are determined by the parameter *κ* (see Method).

To test the hypothesis of a failure in top-down and bottom-up interactions in schizophrenia, we simulated functional disconnection between levels of the hierarchical network. Specifically, connective weights between the higher level intention/goal and the lower level sensorimotor interaction, which are expected to represent altered synaptic connectivity in brain, were slightly modified by adding random noise. We then assessed changes in the robot's behavior and corresponding neural network activity while varying the level of network disconnection (see Methods). Changes in prediction error and robot behavior associated with various levels of disconnection are summarized in Figure 3.

When the level of disconnection was mild, the robot was able to generate outwardly normal behavior. However, due to the impairment of the forward model induced by the functional disconnection, spontaneous intermittent increases of prediction error were observed (Fig. 2B, Fig. 3A), resulting in the robot's intention state being automatically modulated to minimize prediction error. Moreover, intermittent increases in prediction error generation sometimes resulted in irregular switching of the intention state of the network (arrows in Fig. 2B, Movie S2).

When we modeled severe disconnection of the network, the behavior of the robot became disorganized and behavioral sequences no longer followed logical rules (Fig. 3B). We observed abnormal patterns of behavior that are characteristic of more severe cases of schizophrenia, such as cataleptic (stopping or freezing in one posture) and stereotypic (repeating the same action many times) behavior (Fig. 2C, 2D, Movie S2). Modeling experiments indicated these abnormal patterns of behavior appeared as a consequence of the network dynamics converging to a stable equilibrium (limit cycle or point attractor) through the process of the robot attempting to minimize prediction error due to aberrant modulation.

## Discussion

In this study, we demonstrate that schizophrenia can be understood as a failure of essential mechanisms for adaptive behavior. Specifically, mild disconnection in network connectivity resulted in the spontaneous appearance of uncompensated prediction errors and altered interactions within the hierarchical network without external changes in behavior. Based on these findings, we propose that despite no external sensory perturbation in schizophrenia patients, such covert fictive prediction error signals could signal equivalently to normally-generated prediction error signals and, in principle, be indistinguishable by the patient to prediction errors generated from real external sensory stimuli. The results raise the possibility that, in schizophrenia, uncompensated modulatory signals resulting from relatively mild functional disconnection within patients hierarchical neural networks may induce the perplexing feeling that ‘something is wrong’ although s/he cannot identify the source. This feeling, referred to as a delusional mood, is a characteristic prodromal or mild symptom of schizophrenia [27]. In cases where aberrant prediction errors resulted in covert irregular switching of the intention/goal, these results are consistent with the induction of a patient's feeling that their actions are affected by some outside force, termed a disturbance of self. If uncompensated modulatory prediction error signals without explicit external sources are spontaneously generated and cascaded through the network, invading neural circuits related to various perceptual or cognitive modalities, the patient might develop delusions and hallucinations, symptoms observed in typical cases of schizophrenia [28]. At more severe levels of disconnection, we observed overt behavioral defects showing striking similarities to those observed in advanced chronic schizophrenia patients. Thus, the current study provides a systems-level computational principle explaining both the variability and severity of symptoms in schizophrenia, namely the strength and location of connectivity deficits between different layers of a hierarchical network. This idea is consistent with the previously proposed hypotheses of schizophrenia emphasizing the importance of prediction error and functional disconnection in hierarchical networks [10]–[12], [19]–[21], [29].

The present study, for the first time, experimentally links functional deficits in connectivity to the hypothesized role of forward models in schizophrenia. Several studies demonstrate that sensory prediction is impaired in schizophrenic patients [30], [31] while other studies suggest that schizophrenic patients can normally solve a task which requires action control based on forward sensory predictions [32], [33] or that the impaired forward model hypothesis is inconsistent with clinical observations that patients experience an abnormal sense of self only sporadically [34]. Our findings may clarify these puzzling observations by showing that functional dissociation defects in hierarchically organized forward models can be spontaneously induced by network disconnection. We demonstrate that impairments in forward models occur sporadically as a result of intermittent increases in prediction error generation upon network disconnection and triggering a failure in communication between levels of the hierarchical network. On the other hand, forward models corresponding to sensorimotor interaction levels appeared to be preserved.

The present study also links an error-driven behavioral adjustment process to the development of core symptoms of schizophrenia. As known error-detection and error-based behavioral adjustment networks, including medial prefrontal cortex, inferior parietal cortex and temporal parietal junction [35]–[37] are also involved with attribution of agency and self-other referential processing [36], [38] those brain regions may contribute to the pathology of schizophrenia. Studies have suggested that schizophrenic patients have functional abnormalities in these brain regions [20], [37], [38], however, their contribution to the development of clinical schizophrenic symptoms has remained unclear. The spontaneous generation of prediction errors we observed may provide a theoretical framework for linking discrete brain regions and their underlying network computational principles to the development of core symptoms of schizophrenia.

Our results demonstrate that variable symptoms of schizophrenia including covert altered subjective experiences and overt abnormal behavior can be understood as maladaptive processes induced by disconnection between levels responsible for goal-oriented behavior in hierarchical networks. At a systems computational level, the hypothesis of abnormal patterns of behavior as compensation for a failure of brain networks to maintain prediction error equilibrium may provide possible insight into other psychiatric diseases considered to have defective error-related signaling and functional disconnection such as autism [39], obsessive compulsive disorder [40] and attention-deficit/hyperactivity disorder [41].

Our results also support the general premise that in normal brain the minimization of prediction error may comprise a general computational rule of network communication [4]–[6]. Specifically, our model show that production, recognition and learning of adaptive behavior can be achieved based on a single computational principle of minimizing prediction error. This idea is parallel to another line of theory using statistical formulation such as the active inference [42] and predictive coding [4].

Recent studies have emphasized the importance of employing dynamical systems perspectives for understanding higher cognitive functions of the brain [43], [44]. Our findings clearly indicate, for the first time, emergent network properties that produce unexpected effects in underconnected networks on error signaling and abnormal behavior. Our findings may open the door to the further study of critical systems-level issues that should be addressed in future patient and animal model studies. We suggest that our methodology of a network model-driven robot could become an effective approach for examining the hypothesis of network dysfunction and abnormal behaviors in neuropsychiatric conditions. Likewise, physiologists studying the basis of disrupted goal-oriented behavior in animal models and humans may be able to employ similar models to track parameters related to spurious error signals.

## Methods

### Experimental environment

A humanoid robot was used in the role of a physical body interacting with its actual environment. The robot is roughly 50 cm in height, with an arm span of about 30 cm. The robot was fixed to a stand, with tasks involving only movement of the head and arms of the robot. Each arm moves with 4 degrees of freedom (3 shoulders and 1 elbow) and the head motor moves with 2 degrees of freedom (vertical and horizontal). The joints of the robot have a maximum rotation that ranges from 70 degrees to 110 degrees, depending on the type of joint. Rotation ranges were mapped to values ranging from 0.0 to 1.0. Encoder values of these arm joint sensors were received as the current proprioceptive sensory feedback and sent to the network. A vision system mounted on the robot's head automatically fixated a red mark on the object, regardless of the robot's actions. The direction of the robot's head, indicated by encoder values of two neck joints, expressed the object position in the visual field relative to the robot. This relative location of the object was treated as visual input to the network. When the robot received target joint angles, it automatically generated movements corresponding to these angles using a preprogrammed proportional-integral-derivative (PID) controller. Computational processes of the neural network model were implemented in a separate computer communicating with the robot by sending target joint angles and receiving encoder values through a local computer network.

A workbench was set up in front of the robot, and a cubic object (approximately 9×9×9 cm) placed on the workbench served as the goal object. The object was located at two different positions (positions right (R) and left (L)) whose distance was 8 cm.

### Model overview

Inputs to the system were the proprioception 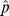 *_t_* (8 dimensional vectors representing the angles of arm joints) and the vision sense 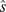 *_t_* (2 dimensional vector representing object position). Based on the current 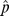 *_t_* and 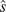 *_t_*, the system generated forward predictions of proprioception *p_t+1_* and the vision sense *s_t+1_* for the next time step. This prediction of the proprioception *p_t+1_* was sent to the robot in the form of target joint angles in generating movements and interacting with the physical environment. Changes in the environment were sent back to the system as sensory feedback (Fig. 1B).

The main component of the system was modeled by a continuous time recurrent neural network (CTRNN). The CTRNN is a type of RNN which implements a feature of biological neurons, namely that the activities of neurons are determined not only by current synaptic inputs but also by the past history of neural states. The current model is considered consistent at the level of the macro-level mechanisms of biological neural systems [24]–[26]. However, consistency in physiological details, such as features of neural activity at the level of individual neurons and characteristics of individual synapses, are not considered in detail. Due to the level of modeling, possible implications of the current results can be discussed only at an abstract level, in terms of the model employed in the current study.

In the current study, the CTRNN is made up of two different types of context units (*fast* and *slow*), each with its own distinct time constant *τ* (“multiple timescale recurrent neural network (MTRNN)” [3]). Through the introduction of multiple timescales, a functional hierarchy, within which the fast sensorimotor units represent “behavioral primitives” (reusable parts of actions) as a lower level and the slow context units represent orders and combinations of primitives as a higher level, can be self-organized [3].

In the proposed model, several slow context units were assigned as “parametric bias” (PB) unit [5]. The PB is static vector input to the network which acts as the bifurcation parameters of nonlinear dynamical systems [5]. Owing to this characteristic of the PB, the proposed network was able to generate multiple patterns of visio-proprioceptive sequences through the self-organized associations between a specific PB activity and different dynamic patterns in the slow context units (i.e. different combinations of behavior primitives). Therefore, PB activity associated with a particular dynamics of the slow context units can be thought of as corresponding to top-down “intention/goal” for a particular task behavior.

In addition to the top-down forward prediction, in order to achieve quick adaptation to environmental changes, we also introduced a bottom-up modulation process [5]. When there is unpredictable change in the environment, a discrepancy between prediction and reality (prediction error) would arise. Based on this prediction error, PB activity is automatically updated in a direction that minimizes prediction error. As a result of this iterative process of bottom-up modulation, PB activity eventually reaches a particular state that corresponds to another task behavior suitable to a new situation, resulting in the robot's ability to flexibly switch its behavior. This switching of the intention through bottom-up modulation can be thought of as corresponding to recognition of a situation. In the generation of behavior, interactions between top-down intention and bottom-up recognition are conducted in real-time, allowing the robot to successfully generate adaptations to unpredictable sensory perturbation.

### Forward dynamics

The neuronal model is a conventional firing rate model, in which each unit's activity represents the average firing rate over a group of neurons. The continuous time characteristics of the MTRNN are described as follows,
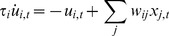
(1)where *u_i,t_* is the membrane potential and *x_i,t_* is neural state of the *i*th unit at time *t*, *w_ij_* is synaptic weight from the *j*th unit to the *i*th unit. Forward predictions of sensory states were made possible by the capacity of the MTRNN to preserve the internal state, which enables it to reproduce complex visio-proprioceptive sequences. In the MTRNN, context units are divided into two groups based on the value of time constant *τ*. The first group consisted of fast context units with a small time constant (*τ* = 10) whose activity changed quickly, whereas the second group consisted of slow context units with a large time constant (*τ* = 100) whose activity, in contrast, changed much more slowly.

The number of MTRNN units for this study was 142. The first 100 units correspond to input-output units (*O*) which receive external input; their activation values *y_i,t_* correspond to output of the MTRNN. The next 40 units correspond to the context units. Among the context units, the first 30 units correspond to the fast context units (*Cf*), and last 10 units correspond to the slow context units (*Cs*). The remaining 2 units correspond to PB units (*PB*). Every unit of the MTRNN, with exceptions described in the followings, is connected to every other unit, including itself. PB units were only connected to slow context units. Input units were not directly connected to slow context units (if *i*∈*O* ∧ *j*∈*Cs*, or if *i*∈*Cs* ∧ *j*∈*O*, then *w_ij_* is fixed at 0).

### Acquisition of forward dynamics (training)

Training of the network was conducted by means of supervised learning using teaching sequences obtained through tutoring by the experimenter. The conventional back-propagation through time (BPTT) algorithm was used for learning of the model network [45]. The objective of training was to find optimal values of connective weights minimizing sensory prediction error. At the beginning of training, synaptic weights of the network were set randomly, resulting in the network generating random sequences. Synaptic weights were modified based on the prediction error between teaching signals and generated sequences. After many repetitions of this process, the prediction error between teaching sequences and model outputs eventually reached a minimum level. This training process was conducted in an off-line manner, in the sense that the prediction of the visio-proprioceptive sequences were generated by means of so-called “closed-loop” operations in which the current prediction of the proprioception and vision state are used as virtual input for the next time step. Thus the network is able to generate visio-proprioceptive sequences without producing actual movements. In the current study, the BPTT was used not for mimicking the learning process of biological neural systems, but rather as a general learning rule. Interested readers could find details of the MTRNN and learning algorithms described in our previous work [3].

The associations between activities of PB units and a particular pattern of behavior can self-organize through a learning process [5]. This process, however, requires fine tuning of parameters in balancing, for example, the learning rate for PB activity and the learning rate for connective weights. Therefore, to reduce the number of arbitrarily set parameters, PB activities in learning process were arbitrary set by the experimenter at values corresponding to different target behavior sequences. Initial states of the context units are set at small random values, meaning that if PB activity had not been set, the network would not have been able to produce multiple behavior sequences.

### Real-time action generation with top-down and bottom-up interaction

The procedure for the real-time top-down and bottom-up interaction during task execution of the robot was conducted within a time window *h* which moves along the increment of the network time-step. This time-window is necessary to avoid the modulation of PB activity according to short-term sensor fluctuations. In this study, the time window *h* is set to 25.

In the top-down prediction process, based on the PB activity at the current time-step *t* and the context states at time-step *t-h*, visio-proprioceptive sequences corresponding to time-steps from *t-h* to *t* are generated by the “closed-loop” operation. In this closed loop operation, PB activity assumes a constant value. The context states at time-step *t-h* act as initial states for this closed loop operation. Generated prediction of visio-proprioceptive sequences for time-steps from *t-h* to *t* are not actually “prediction” in the literal sense of the word, but are more suitable referred to as re-interpretation or “postdiction” [46], [47] of the past based on the current intention.

In the bottom-up modulation process, prediction error within the time window *h* is calculated according to the following equation 2. Prediction error *pe_t_*, is determined as a KL-divergence between the prediction of the network *y_t_* and actual feedback 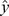 *_t_*,
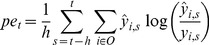
(2)where *O* is a set of indices corresponding to output units. Membrane potential of PB unit is updated in a direction opposite to that of the gradient *∂pe/∂u*, which is also calculated using BPTT algorithm. Actual updating of PB activity is computed according to the following equations:
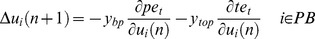
(3)

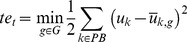
(4)where *n* is an index representing the iteration step in the bottom-up modulation process, the *G* is a set of task behaviors in the rule and 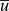 *_k,g_* is a mean activity of PB unit during generation of task behavior *g*, which was used in the training. *γ_bp_* and *γ_top_* are scaling parameters, which were set to 1.0 and 0.0025, respectively. The second term of Eq.3 is an additional term to avoid divergence of PB values in the bottom-up process. *te_t_* is determined as the distance between PB activities for the current time-step and those for the nearest learned behavior. Therefore, the second term of the Eq.3 makes PB activity grow asymptotically toward the values of the nearest learned behavior.

Based on the updated PB activity, top-down prediction of visio-proprioceptive sequences is re-generated. Ideally, the processes of top-down prediction and bottom-up modulation of the PB activity should be iterated many times until PB activity converges. For the current experiment, however, in order to reduce time spent on computation, the number of iterations is limited at 10. After 10 iterations, prediction of proprioception for the time-step *t+1* is generated by the closed-loop operation and is sent to the robot as a target joint angle, along with the increment of the network time-step.

### Simulating functional disconnection

Once a model learns to generate the task behavior, values of synaptic weights are fixed during execution of the robot's behavior and the model network is considered to reproduce the behavior of normal subjects. In the simulation of functional disconnection in the hierarchical network, connective weights between the slow (higher level) and fast (lower level) context units were slightly modified by adding random noise as follows,
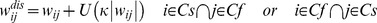
(5)where *U(a)* is the noise following a uniform distribution on the interval [*−a, a*] and *κ* is a parameter determining the level of disconnection. In the mild and severe disconnection conditions, *κ* is set at 0.25, and 0.75, respectively. Adding random noise was applied as one of the simplest implementations for simulating disconnection.

## Supporting Information

Movie S1 **Movie of the robot experiment including (i) flexible switching of behavior through a bottom-up modulation process.** Colored grids indicate neural activity of fast context unit (upper left) and slow context unit (upper right). Color bars indicate neural activity of PB unit corresponding to the intention state. Red line indicates prediction error.
(WMV)

Movie S2 **Movie of the robot experiment including (ii) outwardly normal behavior with aberrant modulation of the intention/goal induced by “mild” disconnection and (iii) stereotypic behavior induced by “severe” disconnection.**
(WMV)
